# Laboratory Experiments on the In Situ Upgrading of Heavy Crude Oil Using Catalytic Aquathermolysis by Acidic Ionic Liquid

**DOI:** 10.3390/ma15175959

**Published:** 2022-08-29

**Authors:** Rima D. Alharthy, Raghda A. El-Nagar, Alaa Ghanem

**Affiliations:** 1 Department of Chemistry, Science and Arts College, Rabigh Branch, King Abdulaziz University, Rabigh 21911, Saudi Arabia; 2 Oil Lab Analysis, Analysis and Evaluation Department, Egyptian Petroleum Research Institute, Nasr City, Cairo 11727, Egypt; 3 PVT-Lab, Production Department, Egyptian Petroleum Research Institute, Nasr City, Cairo 11727, Egypt; 4 PVT Services Center, Egyptian Petroleum Research Institute, Nasr City, Cairo 11727, Egypt

**Keywords:** heavy oil, ionic liquids, catalytic aquathermolysis, asphaltene, SARA analysis

## Abstract

Heavy and extra heavy oil exploitation has attracted attention in the last few years because of the decline in the production of conventional crude oil. The high viscosity of heavy crude oil is the main challenge that obstructs its extraction. Consequently, catalytic aquathermolysis may be an effective solution to upgrade heavy crude oil to decrease its viscosity in reservoir conditions. In this regard, a series of acidic ionic liquids, 1-butyl-1H-imidazol-3-ium 4-dodecylbenzenesulfonate (IL-4), 1-decyl-1H-imidazol-3-ium 4-dodecylbenzenesulfonate (IL-10), and 1-hexadecyl-1H-imidazol-3-ium 4-dodecylbenzenesulfonate (IL-16), were utilized in the aquathermolysis of heavy crude oil. Of each IL, 0.09 wt % reduced the viscosity of the crude oil by 89%, 93.7%, and 94.3%, respectively, after the addition of 30% water at 175 °C. ILs with alkyl chains equal to 10 carbon atoms or more displayed greater activity in viscosity reduction than that of ILs with alkyl chains lower than 10 carbon atoms. The molecular weight and asphaltene content of the crude oil were decreased after catalytic aquathermolysis. The compositional analysis of the crude oil before and after catalytic aquathermolysis showed that the molar percentage of lighter molecules from tridecanes to isosanes was increased by 26–45%, while heavier molecules such as heptatriacontanes, octatriacontanes, nonatriacontanes, and tetracontanes disappeared. The rheological behavior of the crude oil before and after the catalytic aquathermolytic process was studied, and the viscosity of the crude oil sample was reduced strongly from 678, 29.7, and 23.4 cp to 71.8, 16.9, and 2.7 cp at 25, 50, and 75 °C, respectively. The used ILs upgraded the heavy crude oil at a relatively low temperature.

## 1. Introduction

The exploitation of heavy crude oil, and other low-grade oil and gas resources is garnering increasing attention because of the excessive exploitation and rapid consumption of conventional oil and gas resources [1]. The main challenge in extracting heavy crude oil is to reduce its viscosity and improve its fluidity [2].

The chemical composition and physicochemical characteristics of crude oil vary according to its origin. Heavy oil has extremely high viscosity and less API gravity, which render its production, transport, and processing a challenge. Generally, numerous recovery strategies, including thermal, chemical, and biochemical recovery techniques, can be used to optimize its production [3,4,5,6,7]. From these recovery techniques, the aquathermolytic process as a thermal recovery method is the simplest and most effective method that can be used in different processes in enhanced oil recovery (EOR) to produce heavy oil. Therefore, a hot research topic in the petroleum industry is how to maximize heavy crude oil production [8,9,10,11,12]. Heavy oil is chemically composed of saturates, aromatics, resins, and asphaltenes. Moreover, most of the heavy molecules of heavy crude oil mainly contain metal atoms such as nitrogen, sulfur, and oxygen. The use of solvents such as toluene and xylene (high polar solvents that prevent the precipitation of asphaltenes) to improve the steam injection technology can lead to a change in some characteristics of oil, leading to a positive effect on its extraction, transportation, preparation, and processing. On the other hand, the use of solvents with low boiling aliphatic hydrocarbons can coagulate asphaltenes, and clog oily rock pores, which decreases displacement efficiency [13].

The in situ upgrading of heavy crude oil using the catalytic aquathermolytic technique has attracted much attention. Different catalysts such as the Gemini catalyst [14], metal oxide nanoparticles [15], aromatic sulfonate catalysts [8], phosphonate catalysts, and ionic liquids [16] were investigated for the catalytic aquathermolysis of heavy crude oil [13,15]. Metal oxides, especially iron and copper oxides, as potential catalysts for the processes of heavy oil cracking, showed high efficiency during heavy oil aquathermolysis at different temperatures [17]. Bentonite (clay) complex catalysts showed appealing alternative polymer degradation in oil-field wastewater within a wide PH range. They can help in reducing the aromaticity of the polar substances, thereby reducing heavy oil viscosity [18]. Metal oxide nanoparticles also showed high efficiency in the aquathermolytic process. The injection of Fe_2_O_3_ nanoparticles with steam increases the heavy oil production rate, which destroys the C–S, C=C, and C–C bonds of heavy molecules, converting them into lighter ones [15]. In aquathermolysis, the irreversible decomposition of large hydrocarbon molecules into smaller molecules is mainly responsible for viscosity reduction and crude oil improvement [19]. The decomposition process of the hydrocarbon molecules of heavy crude oil cannot occur without the injection of water at a temperature greater than 300 °C. Since these compounds are directly connected with others containing sulfur bonds, the decomposition of the compounds is believed to happen through the breakage of C–S bonds [20]. The general chemical reactions in decomposition processes are believed to use steam to generate carbon dioxide, methane, hydrogen, hydrogen sulfide, and light hydrocarbons with higher molecular weight than that of methane, as shown in Equation (1) [21].

RCH_2_CH_2_SCH_3_ + 2H_2_O ↔ RCH_3_ + CO_2_ + H_2_ + H_2_S + CH_4_ (1)

The released gases from the reaction are vital for the decomposition process; the hydrogen that is released from water reacts with oil through a hydrogen-donating reaction, resulting in viscosity reduction. Kinetic studies on the catalytic aquathermolysis of heavy crude oil are very useful for better understanding the activation energy and reaction mechanisms of the catalytic aquathermolytic process [22,23]. G. Felix et al. reported that the reaction pathways take place in series rather than in parallel under the studied conditions [22,24].

Recently, ionic liquids (ILs) and their applications have attracted great attention due to their advantages of having thermal stability, low vapor pressure, and green characteristics [25,26,27,28,29,30,31]. The catalytic aquathermolysis of heavy crude oil is one of the most popular catalytic applications and was studied by different researchers [8,12,27,32,33,34,35]. Zou et al. studied the effect of IL/H_3_PO_4_ on the degradation and decomposition of asphaltene into low-molecular-weight compounds such as saturates, aromatics, and resins. The IL/H_3_PO_4_ system was also extremely efficient for heavy crude oil desulfurization [36]. Fan et al. synthesized acidic alkyl imidazolium IL BMIM AlCl_4_ and investigated its effect on the viscosity reduction of heavy oil. The results confirmed that the influence of water and sulfur in heavy oil was important in reducing viscosity and upgrading the oil. Moreover, the asphaltene content was reduced to a large extent after the addition of 5% of BMIM AlCl_4_ [37]. Hezave et al. found that the use of dodecyl pyridinium chloride IL in catalytic aquathermolysis drastically reduces the interfacial tension of the crude oil, which in turn decreases the oil viscosity [38]. The experimental results showed that increasing the alkyl chain of the ionic liquid to 12 carbon atoms increased the viscosity reduction of crude oil [38,39]. This may be attributed to the aliphatic interaction between the side chains in both of the ionic liquids and the asphaltene [39]. Saaid et al. compared the performance of the [BMIM][ClO4] IL with that of the chloroaluminate IL as a viscosity reducer of heavy crude oil. The results showed that the chloroaluminate IL was more effective during use in the surface conditions than the [BMIM][ClO4] IL was, which can be used in reservoir conditions because the chloroaluminate IL is more sensitive to pH and moisture [40]. Transition-metal-based imidazolium chloride ILs (R-IL-[FeCl_4_]^−^) were investigated as asphaltene dispersants to upgrade asphaltenic heavy crude oil [25]. The authors used a molecular dynamics simulation (MD) to investigate the compatibility of the ILs with asphaltene and crude oil. Asymmetric dicationic ionic liquids based on alkyl imidazole were evaluated as oil dispersants at different temperatures and showed high efficiency [41].

Ghanem et al. synthesized and characterized a series of imidazolium-based ionic liquids with different alkyl chains (IL-4, IL-10, and IL-16). The surface parameters of the ILs were investigated at different temperatures. The synthesized ILs exhibited excellent thermal and surface properties that facilitated binding them with heavy components under harsh conditions [26]. A new strategy to enhance the flowability of heavy and extra heavy oil was applied here, using these ILs in the catalytic aquathermolysis process. The physicochemical properties and composition analysis of the heavy crude oil before and after the catalytic aquathermolysis process were studied. Moreover, the effect of ILs on the rheological properties of crude oil was investigated. A possible mechanism of catalytic aquathermolysis using imidazolium-based ionic liquids is also proposed.

## 2. Experimental

### 2.1. Materials and Characterization

**Scheme 1:**
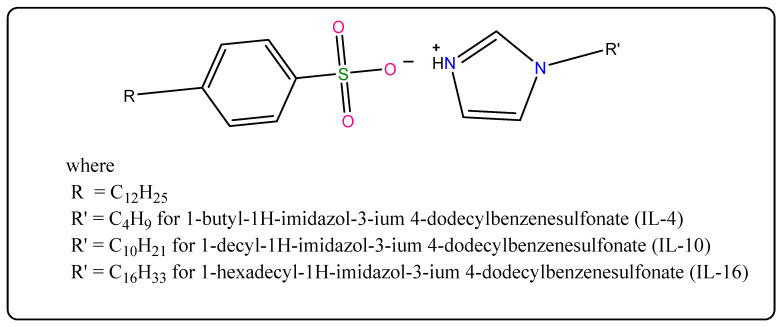
Structure of the used ILs in the aquathermolytic process.

Toluene ≥ 99% analytical reagent; n-heptane ≥ 99% analytical reagent, benzene ≥ 99% analytical reagent, dodecyl benzene sulfonic acid (≥99%), 1-chlorohexadecane (≥99%), 1-chlorodecane (≥98%),1-chlorobutane (≥99%), and imidazole (≥99%) were all purchased from Merck (Darmstadt, Germany). Three different modified imidazolium-based ionic liquids (Scheme 1) were prepared and characterized with the method described elsewhere [26]. In brief, an equivalent amount of 1-alkyl imidazole with different chain lengths (4, 10, and 16 carbon atoms in the alkyl chain) was refluxed with dodecyl benzene sulfonic acid at 80 °C and stirred overnight [26]. The prepared compounds were named for R’ equals to C_4_H_9_, C_10_H_21_, and C_16_H_33_ as IL-4, IL-10, and IL-16, respectively.

The oil samples used in this study were provided by the General Petroleum Company (GPC). The physical properties of the oil sample were measured according to different standard methods, where the density value at 15.6 °C was equal to 0.9651 g/mL, with expanded uncertainty equal to ±0.002 g/mL. Density was measured according to a standard method, ASTM D 4052. The kinematic viscosity of crude oil is equal to 1820.35 cSt at 40 °C according to ASTM D 445 [42], with an expanded uncertainty of 0.25%. The sulfur content of the oil sample, measured according to ASTM D-4294, was 23,520 ppm with an expanded uncertainty of 0.05% [43]. The crude oil sample had a water content of 7.5% ± 1%, which was determined according to standard method ASTM D4006 [44], while the values of the group composition analysis (SARA) are reported in Table 1 according to ASTM D2007 [45].

FT-IR bands were identified in the range of 4000–400 cm^−1^ on the American Nicolet Ia-10 spectrometer using KBr discs supplied by Thermo-Fisher Scientific (Waltham, MA, USA). The kinematic viscosity of the oil samples was measured with a Stabinger viscometer (SVM 3001) provided by Anton Paar (Graz, Austria). Density was conducted through Anton Paar DMA 4100 M with an expanded uncertainty of 0.002 g/mL. The compositional analysis of the crude oil before and after aquathermolysis was determined using a Clarus 500 Perkin Elmer Gas Chromatograph connected with a flame ionization detector (FID) using a selective PIONA capillary column of 100 m in length and 0.25 mm internal diameter (Waltham, MA, USA). Suitable sample capacity was injected into the split/split less injector through a microsyringe according to both the response and linear range of the FID detector. Helium was used as a carrier gas at its optimal flow rate. The system enables the detection of the composition up to C_36_^+^. Cryettee molecular weight (Natick, MA, USA) was used to determine the crude oil’s molecular weight before and after aquathermolysis. A 2 mL solution of the crude oil and benzene was added to the apparatus to detect the freezing point of the solution (ΔT). The average molecular weight of the crude oil can be measured from ΔT as follows [46]:

(2) $$\mathrm{MW}=\frac{1000\times K\times U}{\Delta T\times V}$$

where K is the molal freezing point depression constant, U is the weight of crude oil in grams, ΔT is the freezing point depression in °C, and V is the weight of benzene in grams.

Anton Paar’s (Graz, Austria) MCR 102e was used to study the rheological behavior of the crude oil samples.

### 2.2. Experimental Work on Catalytic Aquathermolysis of Heavy Crude Oil

Table 2 and Table 3 show the orthogonal design of the conducted experiments in this study. We designed the orthogonal and blank experiments to evaluate the performance of the used ILs.

The experimental evaluation of the used ILs was conducted in a Parr autoclave supplied by Parr Instrument Company (Moline, IL, USA). The autoclave volume was up to 500 cc, while the pressure and temperature were up to 100 bar and 350 °C, respectively. The operating conditions (pressure and temperature) were higher than reservoir conditions and were adjusted with the controller unit. The reactor was filled up to only 150 cc with the samples and then operated according to the designed experiment. At the end of the reaction time, the produced oil was left to rest until the temperature had become ambient. Then, the viscosity reduction in the treated oil sample was identified using Anton Paar MCR 102e and according to Equation (3) [47].

(3) $$\Delta \mu =\frac{\mu_{o}-\mu }{\mu_{o}}\times 100$$

where ∆μ % is the viscosity reduction percentage, μ_o_ is the initial viscosity of the oil sample before aquathermolysis in mPa.s, and μ is the final viscosity of the oil sample after aquathermolysis in mPa.s.

**Scheme 2:**
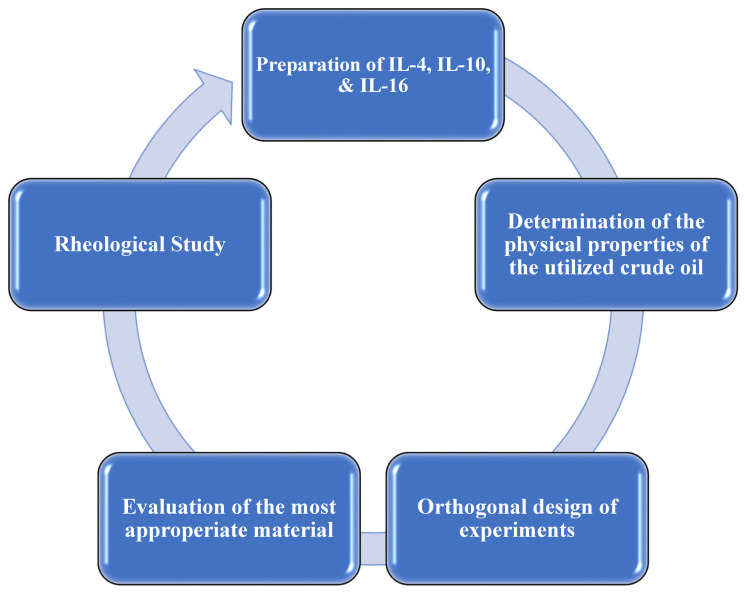
Flowchart of the main steps in this work.

Group composition analysis (SARA) was conducted for the crude oil before and after the aquathermolytic process of the crude oil at optimal conditions. In addition, FT-IR was conducted for asphaltene extracted from the best experimental conditions and compared with the original asphaltene before aquathermolysis. The molecular weight of the crude oil before and after using the ILs was determined with a Cryettee molecular weight apparatus by using Equation (2). Scheme 2 comprises the main steps conducted in this research.

## 3. Results and Discussion

The production of heavy oil is a serious issue in the petroleum industry due to its high viscosity [48,49]. Therefore, it is important to employ effective techniques to in situ decrease crude oil viscosity. Although the high performance of ionic liquids is well-known, their high cost is still a great challenge in field applications compared with organic solvents and other commercial catalysts. The operating conditions can enhance the ability to use ionic liquids in the field because a small amount of ionic liquid can enhance crude oil at a relatively low temperature. Moreover, ionic liquids can dissolve in both water and organic solvents. In general, a full feasibility study is recommended before using ionic liquids in the field.

The results of this study can be categorized into two main sections. First, the catalytic aquathermolysis of heavy crude oil with and without the prepared ILs was conducted according to the designed orthogonal and single-factor experiments. Second, the crude oil after each run was evaluated to obtain the optimal conditions, and heavy oil fractions were investigated.

### 3.1. Catalytic Aquathermolysis of Heavy Crude Oil

The results of the designed orthogonal and single-factor experiments show that the used ionic liquids (ILs) had catalytic effects in the aquathermolytic process. The experiments showed a viscosity reduction of 89.8, 94.3, and 93.7% for ILs (IL-4, IL-10, and IL-16, respectively) under the optimal test conditions. After each experiment, the viscosity of the heavy crude oil with the ILs was measured, and the viscosity reduction was calculated according to Equation (2) in order to evaluate the used ILs as shown in Table S1. IL-10 showed the largest decrease in viscosity at W/O of 3/7, IL concentration = 0.09 wt %, and temperature of 200 °C after 36 h. This was due to the effect of water as a hydrogen donor on cracked crude oil. On the other hand, the same run was conducted as a blank without adding the IL while the other factors were kept constant. The viscosity reduction of the blank test result was 28.5%. The effect of adding the ionic liquid to reduce the viscosity of heavy crude oil in catalytic aquathermolysis is interesting because an ionic liquid is environmentally friendly and has low vapor pressure [29,50,51,52,53]. Viscosity reduction values according to a series of experiments with different conditions are reported in Table S1. The comparison of the viscosity reduction results obtained with and without the ILs reveals that the used ILs may be effective and universal.

The effect of temperature on the viscosity reduction of crude oil can be observed from Table S1. Viscosity reduction increased, sulfur content decreased, and the temperature of the reaction increased. When the temperature increased from 125 to 175 °C, the viscosity reduction increased from 34% to 89% after using IL-10, while the viscosity reduction of IL-16 and IL-4 increased from 34% to 88% and from 32% to 72%, respectively. This was due to increasing temperature increasing the decomposition of heavy components to light components. When the temperature increased above 175 °C, the change in viscosity reduction slightly changed. This was attributed to the cleavage of the alkyl chain of the IL [54]. The temperature range from 150 to 175 °C was optimal for catalytic aquathermolysis using the prepared ILs.

Moreover, the results reveal that the concentration of ILs greatly impacts the catalytic aquathermolytic process, as shown in Table 4. The viscosity reduction increased from 25 to 39, 69, and 89 after using 0.09 wt % of IL-16 at different temperatures (125, 150, 175, and 200 °C). The increase in IL percentage increased the viscosity reduction of the crude oil by a low value for the same IL. This may have been due to ILs with long alkyl tails interacting well with the heavy components, and being able to easily form a steric hinderance to the asphaltene molecules and disperse the asphaltene agglomerates [39]. Moreover, asphaltene dispersion could occur due to the Lewis acid sites in the ILs having the ability to form π–π* interactions with the basic ends in asphaltene molecules. On the other hand, the imidazole rings in ILs are able to form complexes with heavy metals in the asphaltene agglomerates. This could easily decrease the viscosity of heavy crude oil.

**Figure 1:**
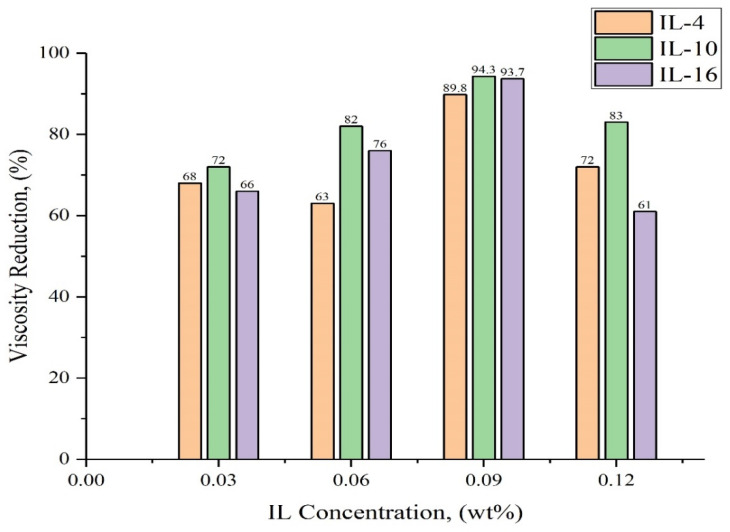
Effect of the concentration of the ILs on crude oil viscosity at 200 °C.

The viscosity reduction of crude oil using different concentrations of the prepared Ils at 200 °C is shown in Figure 1. The optimal concentration of the ILs was 0.09 wt %, while a higher concentration (0.12 wt %) showed a lower viscosity reduction for the three ILs. This may have been due to twisting of the alkyl chains of the prepared ILs at higher concentrations, which decreased their connectivity with the heavy components in the crude oil.

Heavy oil reservoirs contain a certain amount of formation water, which has a significant effect on the viscosity of heavy oil. The combination of water and asphaltenes may lead to the formation of an emulsion, as the asphaltene acts as a natural surfactant. Viscosity is governed by the type of emulsion, where the viscosity of a water in an oil emulsion (W/O) is greater than the viscosity of the crude oil itself, and the viscosity of oil in a water emulsion (O/W) is smaller than the viscosity of the crude oil. In this study, the effect of the water amount on the viscosity reduction of heavy oil by ILs was studied. As shown in Table S1, the mass fraction of water ranged from 20 to 50%, and the best result of viscosity reduction was at 30% water.

### 3.2. SARA Analysis of Heavy Oil

The SARA components of the heavy crude oil before and after the aquathermolytic process are listed in Table 4. Using the ILs in the aquathermolytic process obviously changed the SARA components, where the heavy component contents decreased, and the light ones increased. The saturates and aromatics increased after aquathermolysis and catalytic aquathermolysis using IL-10 from 14.4% and 37.2% to 16.6% and 37.6%, and 21% amd 40.6%, respectively. The asphaltenes and resins decreased from 19.8% and 28.5% to 18.5% and 27.3% after aquathermolysis, and down to 12.8% and 25.6% after using IL-10. This reveals that parts of asphaltenes and resins were cracked, and converted into aromatics and saturates [35]. This proves the activity of the used ILs in the aquathermolysis of heavy crude oil. Khalil et al. reported that aromatic systems could interact largely with the surface of ionic liquid compounds through hydrogen bonding, π bonding, and Van der Waals forces [55]. Such interactions could distort and destabilize the aromatic system, and elongate C–H and C–C bonds. Then, some parts of the large hydrocarbons are destabilized and decomposed into lighter components.

A comparison between different materials that were previously used in the catalytic aquathermolysis of heavy crude oil and the prepared ILs is shown in Table 5 to reveal the effectiveness of the ILs. It is obvious that using the prepared ILs in catalytic aquathermolysis is very promising, since the viscosity reduction percentage was very high (94.3%) with reference to the reasonable applied temperature. In addition, the reduction in heavy components such as asphaltene and resin was considerable.

### 3.3. Effect of Catalytic Aquathermolysis on Compositional Analysis

**Figure 2:**
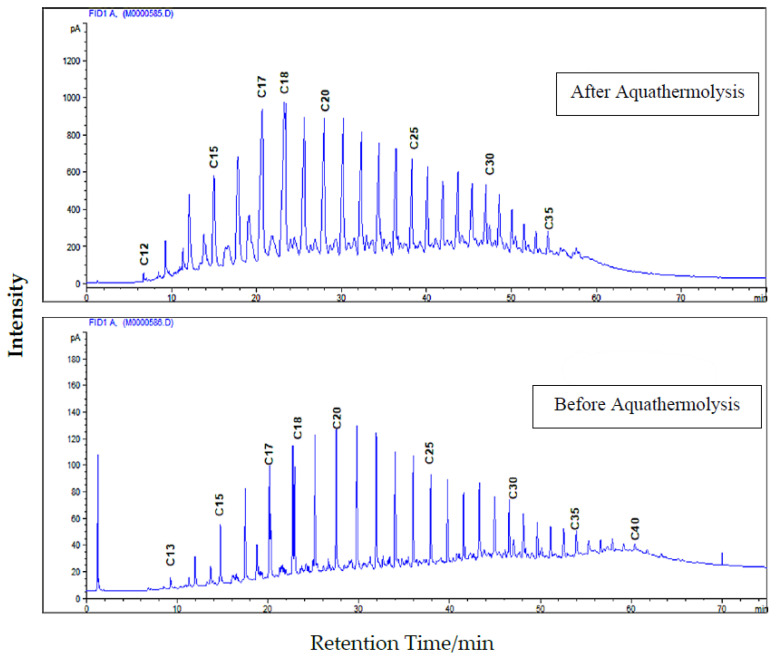
Gas chromatograms of crude oil before and after catalytic aquathermolysis using IL-10.

According to data from Table 4, the catalytic aquathermolytic process decreased the content of heavy hydrocarbons such as asphaltenes and resins, while the light hydrocarbon content (aromatics and saturates) increased. This was proved by the compositional analysis as shown in Figure 2, where the contents of saturated hydrocarbons and aromatics were increased. This may have been due to the cracking of the alkyl chain in the resins and asphaltenes [58]. The molar percentage of the crude oil before and after catalytic aquathermolysis is listed in Table 6. The new lighter components were obviously formed after the catalytic aquathermolytic process. Moreover, there was an increase in the mole percentage of light hydrocarbons from tridecanes (C_13_) to isosanes (C_20_) by 26 to 45%. The mole percentage of the higher carbon numbers, on the other hand, was decreased. Lastly, the heaviest components (C_36_^+^) disappeared in the treated sample, and a new, lighter component (dodecanes) appeared, which confirmed the decomposition of the heavy components into lighter ones, in addition to the partial destruction of asphaltenes and resins.

### 3.4. Elemental Analysis of Asphaltenes and Resins

The results of elemental analysis before and after the catalytic aquathermolysis of both asphaltene and resin are listed in Table 7. After calculation, the carbon content of asphaltene and resin was about 85.34% and 81%, respectively, before catalytic aquathermolysis. This percentage was reduced to 83.1% and 79.39% after catalytic aquathermolysis, while the hydrogen content of asphaltene and resin was increased from 9.1% to 14.13%, and from 11.5% to 14.8%, respectively. In addition, the contents of oxygen and sulfur in asphaltene and resin were decreased. The reduction in both oxygen and sulfur content promotes both C–O and C–S bond breaking [19].

### 3.5. FT-IR of Asphaltene

**Figure 3:**
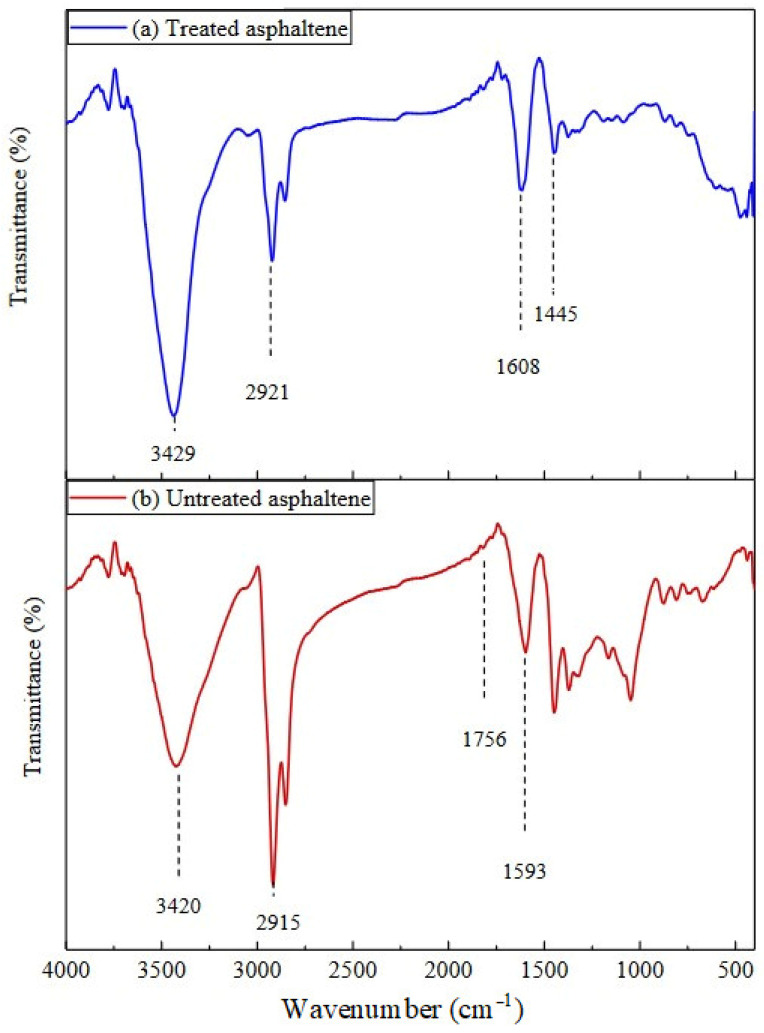
FT-IR spectra of (**a**) treated asphaltenes and (**b**) untreated asphaltenes before and after catalytic aquathermolysis.

Figure 3 shows the FT-IR spectrum of the extracted asphaltene before and after catalytic aquathermolysis. The peaks at 3429 (Figure 3a) and 3420 (Figure 3b) are attributed to the O–H stretching vibration of carboxylic, phenolic, or alcoholic compounds distributed in asphaltene. The typical bands of carbonyl stretching (C=O) for different chemical groups are located in the region of 1800–1600 cm^−1^. The conjugation effect of aryl substitutes shifts the C=O peak to a lower wavenumber, while alkyl substitutes shift to a higher wavenumber. As shown in Figure 3b, there were four observed peaks at 1756, 1709, 1740, and 1689 attributed to the aliphatic ester, aromatic ester, aliphatic carboxylic acid, and aromatic carboxylic acids, respectively. The aliphatic ester and aliphatic carboxylic acid peaks disappeared in the treated asphaltene (Figure 3a). This may have indicated the cleavage of the C–C bond in the long alkyl chain affected by the catalytic aquathermolytic process [59]. Figure 3a shows a peak near 1608 cm^−1^ assigned to the C=C stretching of the aromatic band in the treated asphaltene, and the same peak is appeared at 1593 cm^−1^ in the untreated asphaltene (Figure 3b). This shift may have been due to the high condensation of the aromatic groups in the untreated asphaltene [19].

### 3.6. Rheological Behavior of Crude Oil

**Figure 4:**
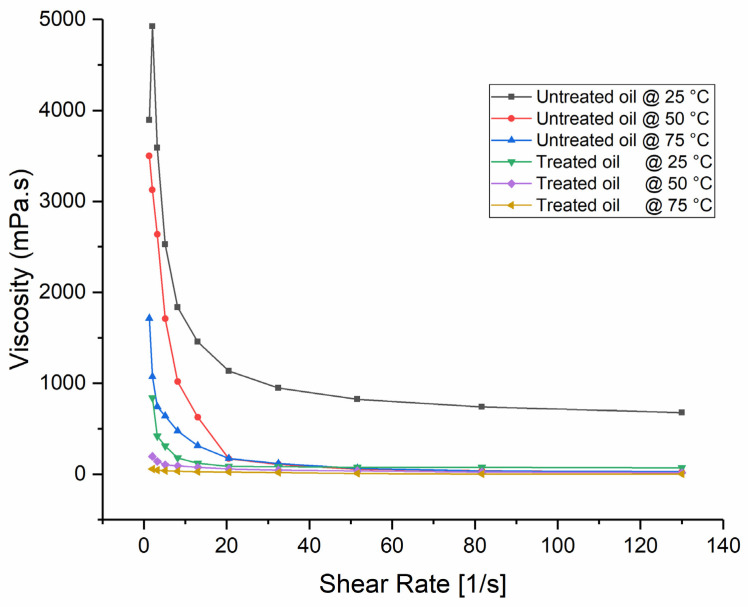
Viscosity flow behavior of crude oil before and after aquathermolysis at different temperatures.

Rheometer MCR 102e was used to investigate the rheological behavior of heavy crude oil by illustrating the viscosity–shear rate relation at different temperatures (25, 50, and 75 °C) as shown in Figure 4. The investigated sample exhibited shear thinning behavior, and the apparent viscosity decreased with increasing temperature. This may have been due to the increase in temperature resulting in a decrease in the agglomeration of the high-molecular-weight components, such as asphaltenes [60]. The objective of the catalytic aquathermolytic process of heavy crude oil is to decrease its viscosity in order to enhance its flowability inside either the reservoir or the pipeline [11]. The effect of catalytic aquathermolysis on the rheological behavior of heavy crude oil at different temperatures is also shown in Figure 4. The results reveal that the viscosity flow behavior of the treated oil was much less than that of the untreated oil. The viscosity of the crude oil sample was reduced strongly from 678, 29.7, and 23.4 cp to 71.8, 16.9, and 2.7 cp at 25, 50, and 75 °C, respectively, after using IL-10 at the optimal conditions. The viscosity curves in Figure 4 show almost Newtonian flow behavior after the temperature reached 50 °C or higher.

**Figure 5:**
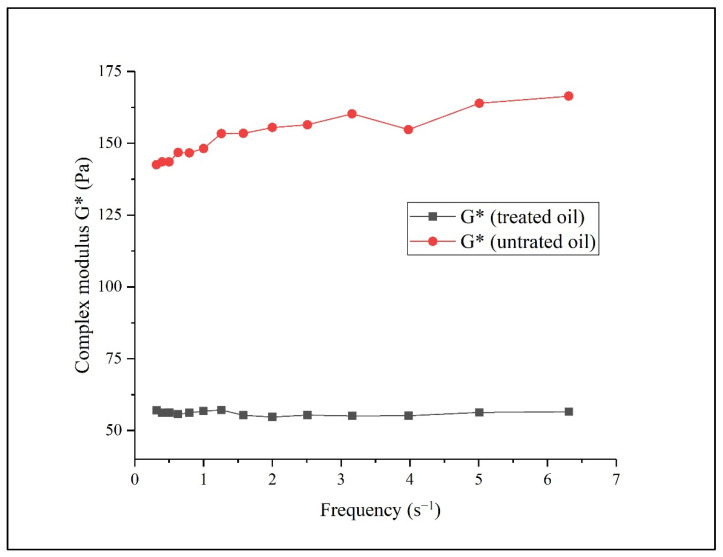
Complex modulus vs. the frequency of the crude oil before and after aquathermolysis at 25 °C.

A viscoelastic study of the treated and untreated crude oil was conducted to illustrate the effect of catalytic aquathermolysis on the rheological behavior of the crude oil. The practical test mainly investigates the effect of oscillating stress or strain on an oil sample. The contribution of recoverable stress energy provisionally stored in the oil sample is elastic or storage modulus G’, while the required energy to initiate the flow is represented by viscus or loss modulus G″. This type of energy is irrecoverable [61,62]. Complex modulus G* is another term that represents the sample resistance against the applied strain. The higher the complex modulus is, the tougher the material. Stress sweep tests over a stress range of 0.1–10 Pa at 1 Hz were conducted to determine the linear viscoelastic range in which complex modulus G* remains constant. Figure 5 demonstrates the frequency against complex modulus G* for heavy crude oil before and after catalytic aquathermolysis using IL-10 at 25 °C. With increasing frequency, the complex modulus of the crude oil sample before catalytic aquathermolysis gradually increased. The complex modulus largely decreased after using IL-10 in the aquathermolytic process. This means that the initiation of the flow was facilitated due to the crude oil’s resistance to the applied strain being decreased. This may have been due to the effect of the ionic liquid on the deformation of the crude oil molecules. All subsequent experiments were conducted at a constant strain of 5% to avoid nonlinear viscoelastic range conditions [61,63].

**Figure 6:**
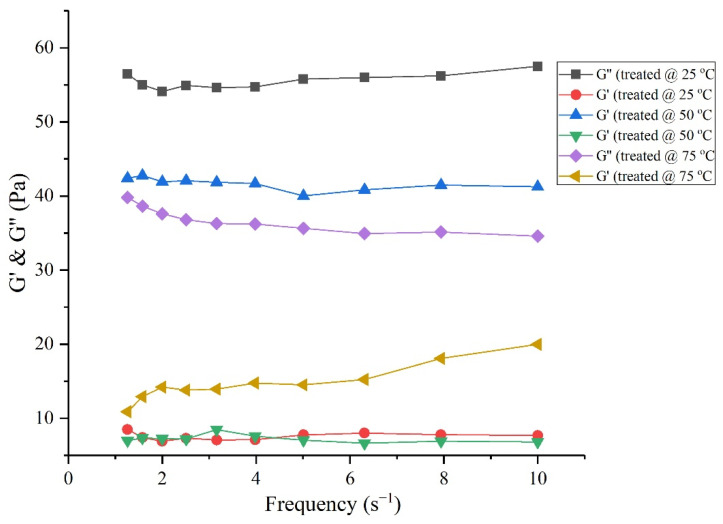
Elastic modulus (G′) and viscus modulus (G″) of the crude oil before and after aquathermolysis at different temperatures.

Heavy crude oil is characterized by a high content of suspended asphaltene that can easily form aggregates and macrostructure networks that are greatly affected by temperature [64,65]. Figure 6 shows the plot of frequency against storage modulus G′ and loss modulus G″ for the crude oil sample before and after the catalytic aquathermolytic process at different temperatures. The results clearly reveal that the crude oil samples behaved like viscus liquid material, where storage modulus G′ had lower values compared with those of loss modulus G″, which means that the energy contained in heavy crude oil is less than the energy wasted in the form of heat. The storage and loss moduli of the treated crude oil by catalytic aquathermolysis decreased significantly compared with the untreated oil sample [65]. This implies that IL-10 could be used in the aquathermolytic process to reduce heavy crude oil viscosity and improve its flowability.

### 3.7. Proposed Mechanism of Catalytic Aquathermolysis

**Scheme 3:**
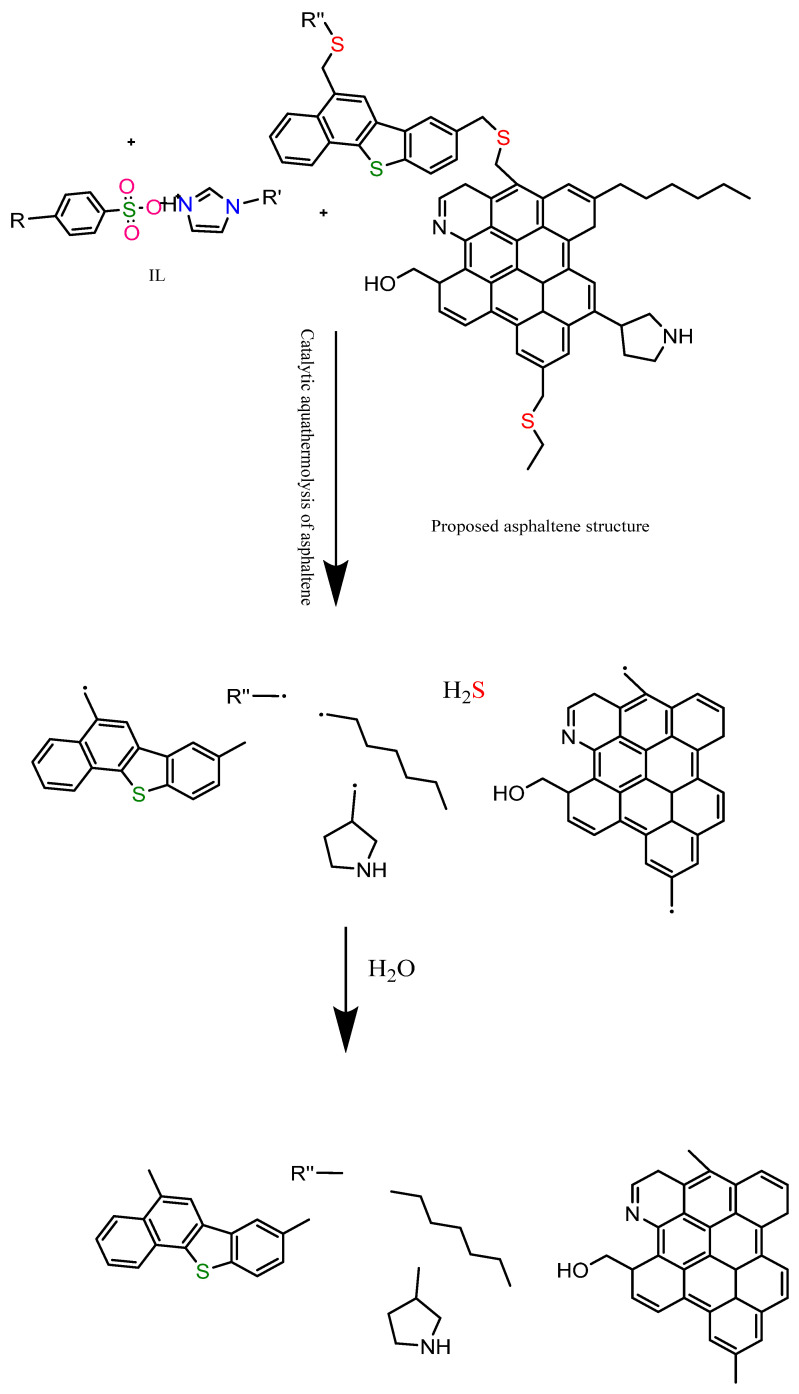
Proposed mechanism of catalytic aquathermolysis of heavy crude oil components (asphaltene).

There are two main approaches to in situ upgrading the viscosity of heavy crude oil [66]. The first is reordering the position of the hydrogen atoms in heavy oil molecules in which hydrogen atoms are moved from heavy parts in the molecule to higher ones to reduce oil viscosity [20]. The formation of coke is a drawback of this pathway. The second approach was first introduced by Hyne et al. [67,68]. It comprises the thermal catalytic cracking of the C–S bond to form different free radical sites, which leads to olefin and coke precursor formation. These free radical sites should be saturated by introducing an external hydrogen source such as water, cyclohexane, or tetralin to the crude oil system in order to produce new molecules with low boiling temperatures. Moreover, heteroatoms such as sulfur and nitrogen can be easily removed. This could increase the hydrogen/carbon ratio and largely reduce viscosity without coke formation. Scheme 3 shows the proposed mechanism for the catalytic aquathermolysis of heavy crude oil using the ILs in the presence of water. π–π interactions between the imidazolium ring and the aromatic ring containing sulfur in the asphaltene molecule are the main step in the desulfurization of the crude oil [69]. In addition, columbic interaction occurs between the IL and the aromatic sulfur compound when the interaction between the cation and anion of IL becomes weaker. This weakness is attributed to the length of the substituted alkyl chain in the cation. Therefore, long alkyl chain ILs were very effective in heavy crude oil desulfurization. The sulfonic group in the IL system could increase the emulsification power of the IL and the interaction between the water and oil to enhance the aquathermolytic process due to the acidic power of the SO_3_H group. Elemental analysis data of asphaltene and resin before and after the catalytic aquathermolysis confirmed the introduction of external hydrogen to the oil system. For these reasons, the heavy components in the crude oil are converted to lighter ones, resulting in a decrease in viscosity and an improvement in oil flowability [54].

## 4. Conclusions

Upgrading asphaltenic heavy crude oil using acidic ionic liquids with different alkyl chain lengths in the catalytic aquathermolysis process was presented in this research. The acidic ionic liquids showed good performance during the process, where the viscosity of the crude oil was reduced to 89, 93.7, and 94.3% after using IL-4, IL-16, and IL-10, respectively. Moreover, the percentages of the heavy component contents (asphaltenes and resins) decreased versus the saturates and aromatics. Gas chromatographic analysis confirmed the conversion of heavy molecules into lighter ones after the catalytic aquathermolytic process. Moreover, the average molecular weight was decreased from 307 to 284 after using IL-10 in the aquathermolytic process. Increasing the efficiency of the IL obviously depended on the alkyl chain length of the IL to a large extent. ILs with long alkyl chains, in addition to the SO_3_H group, such as IL-10 and IL-16, act as resins that have the ability to dissolve asphaltenes in the oil media. The incorporation of acidic ionic liquids into the catalytic aquathermolytic process is very useful for upgrading heavy crude oil due to its high catalytic effect, high thermal stability, and high surface activity.

The rheological properties of crude oil after catalytic aquathermolysis were enhanced, where the crude oil sample behaved like a viscous liquid material with a lower value of G′ than that of G″. These values were decreased significantly in the treated oil sample after using IL-10. These results thus confirm the great efficiency of ILs as catalysts in the aquathermolysis of heavy crude oil. A feasibility study is recommended before using ionic liquids in the field.

## Figures and Tables

**Table 1:** Group composition (SARA) analysis of crude oil.

Chemical Composition, wt.%			
Asphaltenes	Maltene, wt.%		
	Resins	Saturates	Aromatics
19.83	28.524	14.432	37.214

**Table 2:** Orthogonal factors of experiments.

Item	Temperature (°C)	Catalyst (wt %)	Water Concentration (*v*/*v* %)	Time (h)
1	125	0.03	20	12
2	150	0.06	30	24
3	175	0.09	40	36
4	200	0.12	50	48

**Table 3:** Orthogonal design of experiments.

No.	Temperature (°C)	Catalyst (wt %)	Water Concentration (*v*/*v* %)	Time (h)
1	150	0.03	30	24
2	200	0.09	30	36
3	125	0.03	20	12
4	175	0.12	50	12
5	125	0.09	50	24
6	175	0.09	30	36
7	150	0.12	20	48
8	125	0.06	40	48
9	200	0.03	50	48
10	150	0.09	40	12
11	175	0.06	20	24
12	200	0.06	20	12
13	200	0.12	40	24
14	150	0.06	50	36
15	125	0.12	30	36
16	175	0.03	40	48

**Table 4:** Viscosity reduction, sulfur content, and SARA analysis of heavy crude oil before and after aquathermolysis.

Samples	Viscosity Reduction, %	Sulfur Content, ppm	Content, wt.%			
			Asphaltenes	Resins	Saturates	Aromatics
Crude oil	…	23,520	19.83	28.52	14.43	37.21
Aquathermolysis	28.5	22,980	18.5	27.3	16.6	37.6
Aquathermolysis + IL-4	89.8	18,675	14.9	26.8	18.43	39.21
Aquathermolysis + IL-10	94.3	14,580	12.8	25.6	21	40.6
Aquathermolysis + IL-16	93.7	15,120	12.3	26.4	21.5	39.8

**Table 5:** Comparison between previously used catalysts and IL-10 in the catalytic aquathermolysis of heavy crude oil.

Used Catalyst	Catalyst, wt.%	Sulfur Reduction, %	Crude Oil Origin	Viscosity Reduction, %	Asphaltene Content Reduction, %	Ref.
Fe_3_O_4_	5	4.8	Shengli Oilfield	85	21	[56]
Gemini catalyst	0.1	28	Karamay Oilfield	92	6.6	[14]
Fe(C_7_H_7_O_3_S)_3_	0.2	5.2	Y913	90.48	23.6	[57]
Cu(C_7_H_7_O_3_S)_2_	0.15	5.2	Y913	92.19	30	[57]
IL-10	0.09	38	GPC-heavy oil	94.3	35	This work

**Table 6:** Mole percentage of the crude oil before and after catalytic aquathermolysis using IL-10.

		Treated (After Aquathermolysis)	Untreated (Before Aquathermolysis)
Components		Mol. %	Mol. %
Dodecanes	C_12_	0.18	0.00
Tridecanes	C_13_	0.86	0.77
Tetradecanes	C_14_	4.14	2.43
Pentadecanes	C_15_	7.56	4.12
Hexadecanes	C_16_	7.356	4.89
Heptadecanes	C_17_	13.81	10.97
Octadecanes	C_18_	13.44	12.93
Nonadecanes	C_19_	8.93	6.62
Icosanes	C_20_	7.45	6.46
Eneicosanes	C_21_	6.44	7.40
Dodeicosanes	C_22_	4.95	6.69
Tricosanes	C_23_	4.14	5.42
Tetraicosanes	C_24_	3.56	4.83
Petaicosanes	C_25_	2.86	3.80
Hexaicosanes	C_26_	2.66	3.21
Heptaicosanes	C_27_	1.95	2.78
Octaicosanes	C_28_	2.10	3.21
Nonaicosanes	C_29_	1.89	2.66
Tricontanes	C_30_	1.80	2.50
Entricontanes	C_31_	1.28	2.12
Dodetricontanes	C_32_	0.77	1.52
Tritricontanes	C_33_	0.62	1.32
Tetratricontanes	C_34_	0.53	1.11
Pentatricontanes	C_35_	0.44	0.93
Hexatricontanes	C_36_	0.29	0.43
Hepatricontanes	C_37_	0.00	0.32
Octatricontanes	C_38_	0.00	0.26
Nonatricontanes	C_39_	0.00	0.20
Tetracontanes	C_40_	0.00	0.12
Total		100.00	100.00
Mol. Wt.		284.27	307.04

**Table 7:** Elemental analysis of both asphaltene and resin before and after catalytic aquathermolysis.

	C %	H %	N %	S %	O %
Asphaltene, before	85.34	9.1	0.89	1.77	2.9
Resin, before	81	11.5	1.47	1.06	4.97
Asphaltene, after	83.1	14.13	0.46	1.21	1.1
Resin, after	79.39	14.8	0.89	0.82	4.1

## Data Availability

The data that support the findings in the present study are available from the corresponding author upon request.

## Supplementary Materials

The following supporting information can be downloaded at: https://www.mdpi.com/article/10.3390/ma15175959/s1. Table S1: Viscosity reduction values and the sulfur content at each run for the catalytic aquathermolysis process.

## References

[1] Huc A.-Y. (2010). Heavy Crude Oils: From Geology to Upgrading: An Overview.

[2] Li Y.R., Li Q.Y., Wang X.D., Yu L.G., Yang J.J. (2018). Aquathermolysis of heavy crude oil with ferric oleate catalyst. Pet. Sci..

[3] Premuzic E.T., Lin M.S. (1999). Induced biochemical conversions of heavy crude oils. J. Pet. Sci. Eng..

[4] Xia T., Greaves M. (2002). Upgrading Athabasca tar sand using toe-to-heel air injection. J. Can. Pet. Technol..

[5] Pineda-Perez L.A., Carbognani L., Spencer R.J., Maini B., Pereira-Almao P. (2010). Hydrocarbon depletion of Athabasca core at near steam-assisted gravity drainage (SAGD) conditions. Energy Fuels.

[6] Bagci S., Kok M.V. (2001). In-situ combustion laboratory studies of Turkish heavy oil reservoirs. Fuel Process. Technol..

[7] Shokrlu Y.H., Maham Y., Tan X., Babadagli T., Gray M. (2013). Enhancement of the efficiency of in situ combustion technique for heavy-oil recovery by application of nickel ions. Fuel.

[8] Desouky S., Betiha M., Badawi A., Ghanem A., Khalil S. (2013). Catalytic aquathermolysis of Egyptian heavy crude oil. Int. J. Chem. Mol. Eng..

[9] Zhou Z., Slaný M., Kuzielová E., Zhang W., Ma L., Dong S., Zhang J., Chen G. (2022). Influence of reservoir minerals and ethanol on catalytic aquathermolysis of heavy oil. Fuel.

[10] Tirado A., Yuan C., Varfolomeev M.A., Ancheyta J. (2022). Kinetic modeling of aquathermolysis for upgrading of heavy oils. Fuel.

[11] Al-Ghefeili H.M.A., Devi M.G., Al-Abri M.Z. (2022). Magnetite nanoparticle mediated catalytic aquathermolysis of Omani heavy crude oil. J. Indian Chem. Soc..

[12] Saeed A.Q., Al-Zaidi B.Y., Hamadi A.S., Majdi H.S., AbdulRazak A.A. (2022). Upgrade of heavy crude oil via aquathermolysis over several types of catalysts. Mater. Express.

[13] Mukhamatdinov I.I., Salih I.S., Khelkhal M.A., Vakhin A.V. (2020). Application of Aromatic and Industrial Solvents for Enhancing Heavy Oil Recovery from the Ashalcha Field. Energy Fuels.

[14] Chen Y., Yang C., Wang Y. (2010). Gemini catalyst for catalytic aquathermolysis of heavy oil. J. Anal. Appl. Pyrolysis.

[15] Sitnov S., Mukhamatdinov I., Aliev F., Khelkhal M.A., Slavkina O., Bugaev K. (2020). Heavy oil aquathermolysis in the presence of rock-forming minerals and iron oxide (II, III) nanoparticles. Pet. Sci. Technol..

[16] Fan Z.-X., Wang T.-F., He Y.-H. (2009). Upgrading and viscosity reducing of heavy oils by [BMIM][AlCl4] ionic liquid. J. Fuel Chem. Technol..

[17] Ma L., Zhang S., Zhang X., Dong S., Yu T., Slaný M., Chen G. (2022). Enhanced aquathermolysis of heavy oil catalysed by bentonite supported Fe (III) complex in the present of ethanol. J. Chem. Technol. Biotechnol..

[18] Tang Y., Zhou L., Xu Z., Zhang J., Qu C., Zhang Z. (2021). Heterogeneous degradation of oil field additives by Cu (II) complex-activated persulfate oxidation. Environ. Prog. Sustain. Energy.

[19] Chen Y., Wang Y., Wu C., Xia F. (2008). Laboratory experiments and field tests of an amphiphilic metallic chelate for catalytic aquathermolysis of heavy oil. Energy Fuels.

[20] Khalil M., Liu N., Lee R.L. (2017). Catalytic aquathermolysis of heavy crude oil using surface-modified hematite nanoparticles. Ind. Eng. Chem. Res..

[21] Maity S., Ancheyta J., Marroquín G. (2010). Catalytic aquathermolysis used for viscosity reduction of heavy crude oils: A review. Energy Fuels.

[22] Félix G., Tirado A., Al-Muntaser A., Kwofie M., Varfolomeev M.A., Yuan C., Ancheyta J. (2022). SARA-based kinetic model for non-catalytic aquathermolysis of heavy crude oil. J. Pet. Sci. Eng..

[23] Tirado A., Félix G., Kwofie M., Al-Muntaser A., Varfolomeev M.A., Yuan C., Ancheyta J. (2022). Kinetics of heavy oil non-catalytic aquathermolysis with and without stoichiometric coefficients. Fuel.

[24] Félix G., Tirado A., Yuan C., Varfolomeev M.A., Ancheyta J. (2022). Analysis of kinetic models for hydrocracking of heavy oils for In-situ and Ex-situ applications. Fuel.

[25] El-hoshoudy A., Ghanem A., Desouky S. (2021). Imidazolium-based ionic liquids for asphaltene dispersion; experimental and computational studies. J. Mol. Liq..

[26] Ghanem A., Alharthy R.D., Desouky S.M., El-Nagar R.A. (2022). Synthesis and Characterization of Imidazolium-Based Ionic Liquids and Evaluating Their Performance as Asphaltene Dispersants. Materials.

[27] Yunus N.M.M., Dhevarajan S., Wilfred C.D. (2022). Studies on the effect of sulfonate based ionic liquids on asphaltenes. J. Mol. Liq..

[28] Thulasiraman S., Yunus NM M., Kumar P., Kesuma Z.R., Norhakim N., Wilfred C.D., Burhanudin Z.A. (2022). Effects of Ionic Liquid, 1-Ethyl-3-methylimidazolium Chloride ([EMIM] Cl), on the Material and Electrical Characteristics of Asphaltene Thin Films. Materials.

[29] Atef Y., Ghanem A. (2020). Ionic Liquids based on Different Chain Fatty Acids as Green Corrosion Inhibitors for C-steel in Produced Oilfield Water. IOP Conference Series: Materials Science and Engineering.

[30] El-Nagar R.A., Ghanem A.A., Nessim M.I. (2018). Capture of CO2 from Natural Gas Using Ionic Liquids. Shale Gas—New Aspects and Technologies.

[31] El-Nagar R., Nessim M., Abd El-Wahab A., Ibrahim R., Faramawy S. (2017). Investigating the efficiency of newly prepared imidazolium ionic liquids for carbon dioxide removal from natural gas. J. Mol. Liq..

[32] Mukhamatdinov I.I., Khaidarova A.R., Zaripova R.D., Mukhamatdinova R.E., Sitnov S.A., Vakhin A.V. (2020). The composition and structure of ultra-dispersed mixed oxide (Ii, iii) particles and their influence on in-situ conversion of heavy oil. Catalysts.

[33] Chao K., Chen Y., Liu H., Zhang X., Li J. (2012). Laboratory experiments and field test of a difunctional catalyst for catalytic aquathermolysis of heavy oil. Energy Fuels.

[34] Chen Y., Wang Y., Lu J., Wu C. (2009). The viscosity reduction of nano-keggin-K3PMo12O40 in catalytic aquathermolysis of heavy oil. Fuel.

[35] Zhao F., Liu Y., Lu N., Xu T., Zhu G., Wang K. (2021). A review on upgrading and viscosity reduction of heavy oil and bitumen by underground catalytic cracking. Energy Rep..

[36] Zou C., Liu C., Luo P. (2004). Catalytic degradation of macromolecule constituents of asphaltic sand in ionic liquids. J. Chem. Ind. Eng..

[37] Fan H., Zhang Y., Lin Y. (2004). The catalytic effects of minerals on aquathermolysis of heavy oils. Fuel.

[38] Hezave A.Z., Dorostkar S., Ayatollahi S., Nabipour M., Hemmateenejad B. (2013). Dynamic interfacial tension behavior between heavy crude oil and ionic liquid solution (1-dodecyl-3-methylimidazolium chloride ([C12mim][Cl]+ distilled or saline water/heavy crude oil)) as a new surfactant. J. Mol. Liq..

[39] Subramanian D., Wu K., Firoozabadi A. (2015). Ionic liquids as viscosity modifiers for heavy and extra-heavy crude oils. Fuel.

[40] Saaid I., Mahat S.Q.A., Lal B., Mutalib M.I.A., Sabil K.M. (2014). Experimental investigation on the effectiveness of 1-butyl-3-methylimidazolium perchlorate ionic liquid as a reducing agent for heavy oil upgrading. Ind. Eng. Chem. Res..

[41] El Shafiee C.E., El-Nagar R., Nessim M.I., Khalil M.M.H., Shaban M.E., Alharthy R.D., Ismail D.A., Abdallah R.I., Moustafa Y.M. (2021). Application of asymmetric dicationic ionic liquids for oil spill remediation in sea water. Arab. J. Chem..

[42] (2018). Standard Test Method for Density, Relative Density, and API Gravity of Liquids by Digital Density Meter. 2011 ASTM Annual Book of Standards.

[43] ASTM International (2010). Standard Test Method for Sulfur in Petroleum and Petroleum Products by Energy Dispersive X-ray Fluorescence Spectrometry.

[44] (2016). Standard Test Method for Water in Crude Oil by Distillation. ASTM Int 2016.

[45] Khormali A., Moghadasi R., Kazemzadeh Y., Struchkov I. (2021). Development of a new chemical solvent package for increasing the asphaltene removal performance under static and dynamic conditions. J. Pet. Sci. Eng..

[46] Oguamah I.A., Oseh J.O., Yekeen P.N. (2014). Effects of freezing point depression on molecular weight determination of hydrocarbon mixtures. Pac. J. Sci. Technol..

[47] Chen G., Yuan W., Wu Y., Zhang J., Song H., Jeje A., Song S., Qu C. (2017). Catalytic aquathermolysis of heavy oil by coordination complex at relatively low temperature. Pet. Chem..

[48] Aliev F.A., Mukhamatdinov I.I., Sitnov S.A., Ziganshina M.R., Onishchenko Y.V., Sharifullin A.V., Vakhin A.V. (2021). In-situ heavy oil aquathermolysis in the presence of nanodispersed catalysts based on transition metals. Processes.

[49] Hyne J. (1986). Aquathermolysis: A Synopsis of Work on the Chemical Reaction between Water (Steam) and Heavy Oil Sands during Simulated Steam Stimulation.

[50] Buckley J.S. (2012). Asphaltene deposition. Energy Fuels.

[51] Pillai P., Mandal A. (2022). Synthesis and characterization of surface-active ionic liquids for their potential application in enhanced oil recovery. J. Mol. Liq..

[52] Hanamertani A., Pilus R.M., Irawan S. (2017). ICIPEG 2016.

[53] Ma H., Ke H., Wang T., Xiao J., Du N., Yu L. (2017). Self-assembly of imidazolium-based surface active ionic liquids in aqueous solution: The role of different substituent group on aromatic counterions. J. Mol. Liq..

[54] Xu C., Cheng Z. (2021). Thermal stability of ionic liquids: Current status and prospects for future development. Processes.

[55] Khalil R., Chaabene N., Azar M., Malham I.B., Turmine M. (2020). Effect of the chain lengthening on transport properties of imidazolium-based ionic liquids. Fluid Phase Equilibria.

[56] Lin D., Zhu H., Wu Y., Lu T., Liu Y., Chen X., Peng C., Yang C., Feng X. (2019). Morphological insights into the catalytic aquathermolysis of crude oil with an easily prepared high-efficiency Fe_3_O_4_-containing catalyst. Fuel.

[57] Li J., Chen Y., Liu H., Wang P., Liu F. (2013). Influences on the aquathermolysis of heavy oil catalyzed by two different catalytic ions: Cu^2+^ and Fe^3+^. Energy Fuels.

[58] Cui J., Zhang Z., Liu X., Liu L., Peng J. (2020). Studies on viscosity reduction and structural change of crude oil treated with acoustic cavitation. Fuel.

[59] Asemani M., Rabbani A.R. (2020). Detailed FTIR spectroscopy characterization of crude oil extracted asphaltenes: Curve resolve of overlapping bands. J. Pet. Sci. Eng..

[60] Ghannam M.T., Hasan S.W., Abu-Jdayil B., Esmail N. (2012). Rheological properties of heavy & light crude oil mixtures for improving flowability. J. Pet. Sci. Eng..

[61] Kumar R., Banerjee S., Kumar N., Mandal A., Kumar Naiya T. (2015). Comparative studies on synthetic and naturally extracted surfactant for improving rheology of heavy crude oil. Pet. Sci. Technol..

[62] Sakthivel S., Velusamy S. (2020). Eco-efficient rheological improvement of heavy crude oil using lactam based ionic liquids at high temperature high pressure condition. Fuel.

[63] Fan Y., Simon S., Sjöblom J. (2010). Interfacial shear rheology of asphaltenes at oil–water interface and its relation to emulsion stability: Influence of concentration, solvent aromaticity and nonionic surfactant. Colloids Surf. A Physicochem. Eng. Asp..

[64] Taborda E.A., Alvarado V., Franco C.A., Cortés F.B. (2017). Rheological demonstration of alteration in the heavy crude oil fluid structure upon addition of nanoparticles. Fuel.

[65] Montes D., Henao J., Taborda E.A., Gallego J., Cortés F.B., Franco C.A. (2020). Effect of textural properties and surface chemical nature of silica nanoparticles from different silicon sources on the viscosity reduction of heavy crude oil. ACS Omega.

[66] Leon V., Kumar M. (2005). Biological upgrading of heavy crude oil. Biotechnol. Bioprocess Eng..

[67] Kapadia P.R., Kallos M.S., Gates I.D. (2013). A new reaction model for aquathermolysis of Athabasca bitumen. Can. J. Chem. Eng..

[68] Hyne J., Clark P.D., Clarke R.A., Koo J., Greidanus J.W. (1982). Aquathermolysis of heavy oils. Rev. Tec. Intevep..

[69] Ogunlaja A.S., Hosten E., Tshentu Z.R. (2014). Dispersion of asphaltenes in petroleum with ionic liquids: Evaluation of molecular interactions in the binary mixture. Ind. Eng. Chem. Res..

