# Supplementary material for: Laboratory Experiments on the In Situ Upgrading of Heavy Crude Oil Using Catalytic Aquathermolysis by Acidic Ionic Liquid
Source: Materials (Basel). 2022 Aug 29;15(17):5959. doi: 10.3390/ma15175959 (PMC9457198; doi:10.3390/ma15175959)
Supplement: Supplementary file 1 [file materials-15-05959-s001.zip › materials-1827763-supplementary.pdf]

Table S1 viscosity reduction percentage and sulfur content of each run of the catalytic aquathermolysis process

					Viscosity Reduction, %			Sulfur Content, ppm		
**Item**	**Temperature, (ºC)**	**Catalyst, (Wt %)**	**Water concentration, (v/v %)**	**Time, (hrs)**	**IL-4**	**IL-10**	**IL-16**	**IL-4**	**IL-10**	**IL-16**
1	125	0.03	20	12	35	45	49	23155	22150	22810
2	125	0.09	50	24	46	57	61	20950	20585	20940
3	125	0.06	40	48	51	66	71	19590	17980	19850
4	125	0.12	30	36	52	63	68	19250	17540	18450
5	150	0.03	30	24	44.1	55.3	58	22975	20285	20350
6	150	0.12	20	48	59	64.7	62.5	20530	19225	18975
7	150	0.09	40	12	45	57	61	21350	19865	18950
8	150	0.06	50	36	43	61.2	72	20760	17845	18498
9	175	0.12	50	12	65.8	75	78	18280	16580	17345
10	175	0.09	30	36	89	94	93.5	17530	14590	16255
11	175	0.06	20	24	63	71	73	19850	17535	17250
12	175	0.03	40	48	62	69.6	68	19540	18525	17850
13	200	0.09	20	36	89.8	94.3	93.7	16950	15120	15560
14	200	0.03	50	48	68	72	66	18920	18455	18450
15	200	0.06	30	12	63	82	76	17890	16940	16250
16	200	0.12	40	24	72	83	61	17155	18230	18950
